# Surgical leadership in Africa – challenges and opportunities

**DOI:** 10.1515/iss-2018-0036

**Published:** 2019-04-02

**Authors:** Kwabena Frimpong-Boateng, Frank Edwin

**Affiliations:** University of Ghana College of Health Sciences, Accra, Ghana; University of Health and Allied Sciences, Ho, Ghana; Department of Surgery, National Cardiothoracic Centre, Accra, Ghana

**Keywords:** global surgery, leadership, political priority, sub-Saharan Africa, universal health care

## Abstract

Surgical care has been described as one of the Cinderellas in the global health development agenda, taking a backseat to public health, child health, and infectious diseases. In the midst of such competing health-care needs, surgical care, often viewed by policy makers as luxurious and the preserve of the rich, gets relegated to the bottom of priority lists. In the meantime, infectious disease, malnutrition, and other ailments, viewed as largely affecting the poor and disadvantaged in society, get embedded in national health plans, receiving substantial funding and public health program development. It is often stated that the main reason for this sad state of affairs in surgical care is the lack of political will to improve matters in the health sector. Indeed, in 2001, the Commission on Macroeconomics and Health concluded that the lack of political will to sufficiently increase spending on health at the sub-national, national, and international levels was perhaps the most critical barrier to improving health in low-income countries. However, at the root of this lack of political will is a lack of political priority for surgical care.

## Introduction

### The current state of surgical care in Africa

Many have written accounts of the poor state of surgical care and the challenges that surgical leaders in Africa must confront. The general themes include infrastructure and manpower shortages, poor access to surgery, lack of adequate data to facilitate policy making, and many others. The need for surgical services in Africa is enormous, and yet there are insufficient data to point to this situation, which is well known to practitioners living in Africa. Indirectly, this may be inferred from the fact that Africa has the highest percentage per head of surgical disability adjusted life-years in the world [1]. Against this backdrop, it must also be appreciated that the number of surgeons per capita in many African countries is extremely low. Per population of 100,000 residents, Africa has the lowest numbers of specialist surgeons, anesthetists, and obstetricians in the world (0.5, 0.1, and 0.3, respectively) compared to Europe (36.2, 15.7, and 18.2, respectively) [2]. Migration of health workers is contributory to this trend. For example, 68% of Ghanaian-trained medical graduates left the country between 1993 and 2000 [3], and a study of first-year Nigerian medical students found that 76% planned to work overseas once qualified [4]. Not surprisingly, access to specialist surgical care is extremely low in many parts of sub-Saharan Africa. In children born with congenital heart defects, access to surgical or interventional therapy within 2 years of birth is probably <1% in most parts of sub-Saharan Africa [5]. This happens to be the case in the most specialized branches of surgery where shortage of trained manpower, lack of infrastructure, and inadequate health-care financing combine to limit access to surgical services to the point of extinction.

## Global surgery – the Lancet Commission

On the global scene, these same crucial gaps in surgical service delivery and policy are evident. The Lancet Commission on Global Surgery (LCGS) was launched in January 2014 to address these deficiencies on a global scale [6]. The LCGS has noted that many low-middle income countries (LMICs) face a multifaceted burden of infectious diseases, maternal diseases, neonatal diseases, non-communicable diseases, and injuries, and that, in view of the large projected increase in the incidence of surgical diseases in LMICs, the need for surgical services in these regions will continue to rise substantially until 2030 [6]. This forecast is most relevant in sub-Saharan Africa, whose countries are largely of the LMIC extraction. In the case of sub-Saharan Africa and many other LMICs, the LCGS pointed out that deficits in the surgical workforce are often representative of broad challenges in the public sector, and the lack of national policies and strong coordination led by the central government undermines service delivery.

Universal health care (UHC) has emerged as one of the most important policy goals supported by the World Health Organization (WHO), World Bank, United Nations, and many governments in LMICs. The Commission identified four levels of surgical coverage to facilitate the attainment of UHC – the procedures that must be covered; the packages of surgical and anesthesia care in which different procedures are grouped, funded, and purchased; the platforms on which packages of surgical and anesthesia care are delivered; and the national and international policies that assure universal coverage and that inform how this coverage is organized and delivered. Among its policy recommendations to governments and ministries of health and finance, the LCGS indicated that UHC policies should include surgery and cover basic packages of surgical and anesthesia care [6].

In emphasizing the critical role of political influence identified by the LCGS, others have pointed out that the main reason for this sad state of affairs in surgical care is the lack of political will to improve matters in the health sector [7].

Indeed, in 2001, the Commission on Macroeconomics and Health concluded that the lack of political will to sufficiently increase spending on health at the sub-national, national, and international levels was perhaps the most critical barrier to improving health in low-income countries [8]. However, at the root of this lack of political will is a lack of political priority for surgical care and other confounding societal needs.

### Low manufacturing capacity

Political priority setting is perhaps the most important factor in determining the progress of surgical service in Africa. Mention should be made, however, of the lack of manufacturing capability as a determinant of inadequate surgical services on the African continent.

In most African countries, all surgical supplies and instruments, including basic ones such as dissecting and artery forceps, scissors, and clamps, as well as equipment from sphygmomanometers through pulse oximeters to magnetic resonance imaging machines, are imported. The same applies to general consumables including gauze, plaster, syringes, needles, and plaster of Paris.

Spare parts for equipment, such as those from oxygen manufacturing plants, are sometime difficult to obtain. The result is that surgeries can be cancelled due to unavailability of oxygen or lack of simple inputs such as gloves and gauze.

Surgical leaders in Africa have to engage political authorities and the business community to consider investments in medically related industries. It is not an easy undertaking to convince the business community to invest in areas with a long incubation period, relatively low turnover, and that may not have large profit margins as seen in trading activities such as importation and wholesale of food articles such as rice, sugar, and fish. Some business people want assurances from political authorities that items produced will have guaranteed uptake at least by government-controlled health institutions.

Production of even the very basic surgical instruments will involve the establishment of machine tooling factories including Computer Numerical Control machines. This also requires training or engagement of specialized workers, who may not be adequately compensated in Africa. These are areas that most business people shy away from.

In a number of countries, what is required may be a shift of emphasis and reorientation of business models. In countries such as Ghana, Liberia, Nigeria, and Democratic Republic of Congo, where there is significant rubber production, it may not be too much of a problem to replace unprofitable vehicle tire production with the manufacture of surgical gloves, condoms, catheters, and nasogastric tubes, among others.

Many countries also produce cotton for the clothing industries but at the same time import large quantities of gauze from abroad. In such cases, a change in business model will be to use the cotton to produce gauze and cotton wool for hospitals.

It is the belief of surgical leaders in Africa that if importation of medical consumables is significantly reduced, funds will be freed to improve the working conditions and salaries of health personnel and thereby improve surgical service delivery on the continent. Some progress has been made; however, a lot remains to be done in localizing the manufacture of surgical instruments and equipment as well as surgical consumables.

### Political priority for surgical care

The health of the African population and attempts to improve it has become an important concern for many African governments in the recent past. For example, many public health programs have been developed and deployed to address the challenges posed by human immunodeficiency virus (HIV)/acquired immunodeficiency syndrome (AIDS), malaria, and tuberculosis in Africa. These programs have been pursued diligently in several African countries because they have become embedded in the national health plans of these countries. By contrast, the provision of surgical care is neglected or receives very low priority in Africa. This is in spite of the fact that surgical conditions worldwide claim more lives each year than HIV/AIDS, tuberculosis, and malaria combined (16.9 million vs. 3.83 million) [9]. The national health plans of countries are a reflection of the long-term health priorities, plans, and targets of nations for specified periods. In a systematic review of the national health plans of 43 independent sub-Saharan African countries, all countries represented had documented plans and measurable targets for the reduction of HIV and tuberculosis. Of the 4064 health targets identified in these plans, only 2% were related to surgical conditions or surgical care; 33% of the policies had no surgical targets whatsoever [10].

These policy lapses make it evident that the single most important challenge that surgical leadership must address in Africa is generating political priority for the surgical mission across the countries on the continent. Surgical leaders in Africa must articulate a policy that facilitates the placement of surgical care on the national health plans of African countries.

### Why surgical care receives low priority in Africa

Shiffman and Smith’s seminal paper in 2007 [11] posed the question, “Why do some global health initiatives receive priority from international and national political leaders whereas others receive little attention?” The authors were attempting to explain why after two decades, a global initiative to reduce maternal mortality launched in 1987 appeared stuck in an early phase of development, hampered by several difficulties. In their analysis, the authors proposed and applied a framework consisting of four categories: the strength of the actors involved in the initiative, the power of the ideas they use to portray the issue, the nature of the political contexts in which they operate, and the characteristics of the issue itself.

Surgical leaders in Africa have not adequately addressed how the many competing health needs on the continent receive different political priority and resources at the national level, with some becoming embedded in national health policy while some do not. For example, Africa has a high prevalence of heart disease in children and young adults, mostly from congenital heart disease and rheumatic heart disease [12]. In addition, current lifestyle changes in Africa have also contributed to a rising incidence of the metabolic syndrome and its complications [13]. The major components of the metabolic syndrome – dyslipidemia, obesity, hypertension, and dysglycemia – are also major risk factors for ischemic heart disease and cerebrovascular disease. As a result of these changes, a 2013 paper estimated that the age-standardized mortality rates for ischemic heart disease will rise by 27% in African men and 25% in women by 2015, and by 70% and 74%, respectively, by 2030 [14]. In spite of these significant pointers to its public health importance, surgical care of cardiovascular disease receives only a small fraction of the resources devoted to the treatment of infectious disease in Africa. In many African countries, surgical care of cardiovascular disease has little, if any, priority at all.

The insufficient priority in Africa is not limited to the cardiothoracic surgery specialty. Similar concerns are shared by colleagues in pediatric surgery, neurosurgery, and others [15], [16]. Shiffman and Smith’s framework suggests that political priority for surgical care in Africa is low because the proponents of the issue have not communicated a clear policy need using powerful ideas that take advantage of the political contexts of the times.

### Defining the framework

Political priority according to Shiffman and Smith’s framework is the degree to which national political leaders actively give attention to an issue, and back up that attention with the provision of financial, technical, and human resources that are commensurate with the severity of the issue [11]. Political priority is evidently present when political leaders publicly and privately express sustained concern for an issue, national policies are enacted to address the issue, and resources are provided in a measure commensurate with the severity of the issue. Political priority is not the only requirement for the surgical agenda to succeed in Africa. However, the provision of adequate surgical care has little chance of success without attaining political priority.

Shiffman and Smith’s framework [11] consists of four categories: proponents, issue at stake, ideas, and political contexts. In our context, the proponents are the surgical leaders driving the issue at stake (political priority for surgical care), who use certain ideas to portray the issue to the public and political leaders operating in political contexts dictated by the times.

### Using the framework

Shiffman and Smith’s framework has since been widely adopted and used by several workers in the analysis of other global health issues [17], [18]. The same framework is helpful in devising a strategic plan to improve surgical care in Africa.

Proponents of the framework describe the strength of the individuals and organizations concerned with the issue, in this case, political priority for surgical care. Proponents of any health issue are likely to succeed if they operate as a community with strong leadership, a clear cohesive policy, and effective guiding institutions while utilizing grassroots mobilization to press political authorities to address the issue at the national and international levels. For example, the Task Force for Child Survival became the powerful global guiding institution for child survival in the 1980s that inspired civil society mobilization under the strong leadership of Jim Grant of the United Nations International Children’s Emergency Fund [19].

The issue at stake is surgical care and obtaining political priority for it. Surgical care involves nation-specific data on surgical disease burden, surgical workforce capacity, and the infrastructural requirements that facilitate the delivery of surgical care. Surgical leaders in Africa need to provide such data to support advocacy efforts aimed at obtaining political priority.

The ideas proponents use to portray the problem of inadequate surgical care in Africa will determine the degree of buy-in surgical leaders will obtain on the political front. The global surgical community has attempted to portray the case for the surgical mission in several different ways: surgery as an integral component of primary health care; as a cost-effective intervention; as a preventive, not just curative, public health measure; as a public health issue that affects everyone; as an economic imperative; as a necessity for women’s equity and empowerment; and as a human right [19]. Without a unifying powerful idea, it is not surprising that the surgical mission has not attained the priority it ought to. Surgical leaders in Africa must come up with a simple idea easily grasped by the public and political elite to have any chance of effective advocacy. Improving surgical care will require attention to the areas of quantifying surgical disease burden, manpower training, infrastructure development, and health-care financing. The key idea for improving surgical care must capture exactly what surgical leaders require policy makers to do in Africa. The surgical community must agree on the approaches to be used to address the issue of prioritization and portray it in a manner that resonates with political leaders at the helms of resource allocation.

Political contexts involve governance structures and policy windows. The prevailing governance structures regarding health care may provide a platform for effective collective action on a particular health issue. In a somewhat similar manner, governance structures may promote certain policy directions that present a window of opportunity for proponents to influence policy makers. In September 2000, when 189 heads of states gathered at the United Nations in New York and adopted eight human development goals that became known as the Millennium Development Goals (MDGs), three of which were health related, global governance structures had opened policy windows for all manner of health-care proponents. The three health-related MDGs (reduce child mortality; improve maternal health; and combat HIV/AIDS, malaria, and other diseases) represented the most broadly supported health-care aspirations ever established. HIV/AIDS, infectious disease, and maternal and child survival advocates were quick to notice the opportunities presented by the prevailing governance structures and open policy windows. Fifteen years later, remarkable gains had been made in the fight against HIV/AIDS, malaria, and tuberculosis; the under-five mortality rate had declined by more than half; and maternal mortality was down by 45% worldwide [20].

If the MDGs represented a timely alignment of governance structures and opportunity for advocacy, it was a missed policy window for surgical leaders pursuing the surgical mission who argued that surgical care is important for achieving the MDGs but were unable to formally link surgical care and the MDGs in a cohesive policy instrument. Only recently did global surgery advocates succeed in persuading WHO’s Executive Board to pass “Strengthening Emergency and Essential Surgical Care and Anesthesia as a Component of Universal Health Coverage” as a resolution at the World Health Assembly in May 2015 [21].

### Political priority – an illustration

In Ghana, open heart surgery began in 1964 when Professor C.O. Easmon’s team successfully performed closure of an atrial septal defect using surface cooling to achieve hypothermia [22]. When the second patient did not survive the open heart procedure, stiff opposition to further development of open heart surgery in Ghana ensued, and the endeavor literally disappeared from the country’s priority. Attempts to revive the program were initiated in the early 1980s by Kwabena Frimpong-Boateng, a Ghanaian surgeon who had trained in Hannover, Germany. The project concept was initially considered of very low priority by many of the authorities in the country’s medical establishment and some political leaders as well [22]. After prolonged and difficult negotiations with government leaders, the project was accepted in principle. In the language of Shiffman and Smith’s framework, a proponent had sought political priority for the issue of establishing a modern cardiothoracic surgery service in Ghana. The key idea used to portray the issue was the essential nature of the service. The political context at the time probably facilitated the process: the country was then ruled by a military head of state not encumbered by lengthy bureaucracies typical in multi-party parliamentary democracies. Typically, one gets the green light if the head of state is convinced of the priority of the project. That political priority had been attained was evidenced by the fact that by August 1986, official recognition and acceptance of the project by the Ministry of Health had occurred, a training program for nurses and technicians assigned to the project was being implemented in Hannover, and a biomedical engineering company had been tasked to provide estimates and suppliers credit funds for the project [22]. Ghana’s National Cardiothoracic Centre was officially commissioned in April 1992 and is possibly the foremost in the West African region currently, being the only one fully accredited by the West African College of Surgeons for training cardiothoracic surgeons in the region [23].

## ICT and surgical service delivery

The use of mobile phones in Africa has grown rapidly over the last 15 years with mobile network coverage rising from 16% in the late 1990s to >90% of its population in 2011. It is estimated that there are about a billion mobile phones now in Africa. Internet connectivity is, however, only 15%.

This trend is encouraging the surgical leaders in Africa to consider the use of information and communications technology (ICT) in improving surgical services on the continent, with ICT in this context being tools that facilitate communication and the processing and transmission of information by electronic means, especially mobile telephones, computers, and the Internet.

Theoretically, ICT can facilitate online consultations by patients and doctors through websites and email, distance referrals, emergency evacuations, and advance transmission of images and data of patients from remote locations and ambulances. In practical terms, for such a system to be successful, a critical mass of professional and community users of ICTs in health as well as adequate ICT infrastructure has to be available; however, unfortunately, this is not the case in Africa.

The WHO Global Observatory for eHealth series, launched in 2005, examines trends in the uptake of telemedicine, from the well-established to newly emerging telemedicine applications, with an emphasis on the needs of developing countries. The publication is targeted at telemedicine practitioners and policy makers in health and information technology, as well as health-care practitioners interested in adopting telemedicine services. The data gathered thus far indicate that countries in Africa are making improvements in health as a direct benefit of ICT. Also, eHealth innovations such as electronic health records, computer-assisted prescription systems, and clinical databases are transforming health today, and hold even greater promise for the future. There is reasonable expectation that ICTs are addressing challenges of maternal and child health and infectious diseases in rural Africa. Their use in surgical care is, however, very limited.

## Conclusion

Leadership is a cornerstone of improving surgical care in populations. Lack of manufacturing capability with its attendant importation of almost all inputs for surgical care limits effective practice and growth of surgery in Africa. Also, the paucity of ICT infrastructure as well as the lack of critical mass of professional and community users of ICTs in health put the brakes on ICT in surgical practice on the continent. Most important, access to surgery is poorly represented in the current national health plans of many African countries. This low-priority status is the key challenge confronting surgical leaders in Africa. Surgical care is unlikely to significantly improve in Africa unless surgical leaders find innovative ways to get adequate representation of surgery in national health plans. Unfortunately, the private sector in most African countries is not well developed and governments have a virtual monopoly in deciding the level and direction of development. Almost all policy and developmental initiatives emanate from governments. It is therefore crucial that in matters of promotion of surgical service, advocacy is sought at high political levels.

Shiffman and Smith’s model provides a framework by which surgical leaders may devise strategies to address this problem.

## Supplementary Material

The article (iss-2018-0036) offers reviewer assessments as supplementary material.
