# Supplementary material for: Surgical leadership in Africa – challenges and opportunities
Source: Innov Surg Sci. 2019 Apr 2;4(2):59–64. doi: 10.1515/iss-2018-0036 (PMC6754052; doi:10.1515/iss-2018-0036)
Supplement: Supplementary file 1 [file iss-4-20180036-s001.pdf]

Reviewer Assessment

Kwabena Frimpong-Boateng* and Frank Edwin

Surgical leadership in Africa – challenges and opportunities

https://doi.org/10.1515/iss-2018-0036
Received November 8, 2018; accepted March 7, 2019

***Corresponding author: Kwabena Frimpong-Boateng,** University of Ghana College of Health Sciences, Accra, Ghana, E-mail: missiondestiny@yahoo.com

Reviewers' Comments to Original Submission

Reviewer 1: anonymous

Nov 29, 2018

**Reviewer Recommendation Term:**	Accept
**Overall Reviewer Manuscript Rating:**	40
**Custom Review Questions**	**Response**
Is the subject area appropriate for you?	5 - High/Yes
Does the title clearly reflect the paper's content?	5 - High/Yes
Does the abstract clearly reflect the paper's content?	4
Do the keywords clearly reflect the paper's content?	5 - High/Yes
Does the introduction present the problem clearly?	5 - High/Yes
Are the results/conclusions justified?	4
How comprehensive and up-to-date is the subject matter presented?	5 - High/Yes
How adequate is the data presentation?	N/A
Are units and terminology used correctly?	N/A
Is the number of cases adequate?	N/A
Are the experimental methods/clinical studies adequate?	N/A
Is the length appropriate in relation to the content?	5 - High/Yes
Does the reader get new insights from the article?	5 - High/Yes
Please rate the practical significance.	4
Please rate the accuracy of methods.	4
Please rate the statistical evaluation and quality control.	N/A
Please rate the appropriateness of the figures and tables.	N/A
Please rate the appropriateness of the references.	4
Please evaluate the writing style and use of language.	5 - High/Yes
Please judge the overall scientific quality of the manuscript.	3
Are you willing to review the revision of this manuscript?	No: only a few mistakes in spelling and grammer. Someone should look at it. But minor problem.

Comments to Authors:
None

Open Access. © 2019 Frimpong-Boateng K., Edwin F., published by De Gruyter. This work is licensed under the Creative Commons Attribution 4.0 Public License.

Reviewer 2: John Primrose

Nov 30, 2018

**Reviewer Recommendation Term:**	Revise with Major Modifications
**Overall Reviewer Manuscript Rating:**	60

**Custom Review Questions**	**Response**
Is the subject area appropriate for you?	3
Does the title clearly reflect the paper's content?	5 - High/Yes
Does the abstract clearly reflect the paper's content?	5 - High/Yes
Do the keywords clearly reflect the paper's content?	1 - Low/No
Does the introduction present the problem clearly?	4
Are the results/conclusions justified?	4
How comprehensive and up-to-date is the subject matter presented?	3
How adequate is the data presentation?	N/A
Are units and terminology used correctly?	N/A
Is the number of cases adequate?	N/A
Are the experimental methods/clinical studies adequate?	N/A
Is the length appropriate in relation to the content?	2
Does the reader get new insights from the article?	5 - High/Yes
Please rate the practical significance.	4
Please rate the accuracy of methods.	N/A
Please rate the statistical evaluation and quality control.	N/A
Please rate the appropriateness of the figures and tables.	N/A
Please rate the appropriateness of the references.	3
Please evaluate the writing style and use of language.	5 - High/Yes
Please judge the overall scientific quality of the manuscript.	4
Are you willing to review the revision of this manuscript?	Yes

Comments to Authors:
This is a well written and insightful contribution on surgical leadership on the continent of Africa. There are many interesting insights and suggestions which may have impact in difficult political environments. I have some suggestions. I think it is too long and can be edited down without losing much content. The component on the cardiac surgery in Ghana can be reformatted to be less personal and certainly the references as currently configured are not necessary. It is useful as an example however of how progress can be made in adverse circumstances. I am somewhat surprised the Lancet Commission on Global Surgery is not mentioned and I think as this is the document of the moment it ought to be referred to and analysed in the African context. Some key words are needed

Authors' Response to Reviewer Comments

Dec 10, 2018

Many thanks for taking the time to review our paper. Our responses to the queries arising from the review are indicated below.

Reviewer 2:

Query 1. I think it is too long and can be edited down without losing much content.

Response: We have attempted to edit down the script with some success but this has been largely undermined by the inclusion of a new segment on the report of the Lancet Commission on Global Surgery and its relevance to sub-Saharan Africa. The word count is nonetheless at least 200 fewer.

2. The component on the cardiac surgery in Ghana can be reformatted to be less personal and certainly the references as currently configured are not necessary. It is useful as an example however of how progress can be made in adverse circumstances.

Response: We have done this and reformatted the segment using impersonal language.

3. I am somewhat surprised the Lancet Commission on Global Surgery is not mentioned and I think as this is the document of the moment it ought to be referred to and analyzed in the African context.

Response: We have included a new segment describing the relevance of the Lancet Commission report's in the context of surgical leadership in Africa. Thank you for pointing this out.

4. Some key words are needed.

Response: We have now included 5 keywords.

We have indicated the changes in the document using red highlighted fonts. Thank you once again.
